# A novel approach for the metric analysis of fern fronds: Growth and architecture of the Mesozoic fern *Weichselia reticulata* in the light of modern ferns

**DOI:** 10.1371/journal.pone.0219192

**Published:** 2019-06-27

**Authors:** Candela Blanco-Moreno, Bernard Gomez, Jesús Marugán-Lobón, Véronique Daviero-Gomez, Ángela D. Buscalioni

**Affiliations:** 1 Unidad de Paleontología, Departamento de Biología, Universidad Autónoma de Madrid, Cantoblanco, Madrid, Spain; 2 CNRS-UMR 5276 Terre, Planètes, Environnement, Université Lyon 1, Claude Bernard, Villeurbanne, France; 3 Dinosaur Institute, Natural History Museum of Los Angeles County, Los Angeles, CA, United States of America; Indiana University Bloomington, UNITED STATES

## Abstract

The architecture of primary and secondary pinnae of the fossil fern *Weichselia reticulata* (C. Stokes et Webb) Fontaine is studied based on 28 large specimens from the upper Barremian La Huérguina Formation of the fossil locality Las Hoyas, Cuenca, Spain. The study of the specimens is performed through a morphometric analysis consisting in a reformulation of the Branching Algorithms method of shape description including measurements (insertion angle, distance between pinnae, first segment length and rachis width) and ratios (interval ratio, branching ratio and tapering ratio). A protocol to relocate isolated fragments of fossil pinnae is also established using the interval ratio (distance between pinnae/ previous distance between pinnae) and insertion angle of the pinnae. All specimens show a similar architecture, having elliptic primary pinnae with a sinuous apically tapering primary rachis and triangular secondary pinnae with pinnules of different morphologies. The analysis of the architecture allows to propose that the position of the frond was plagiotropic and that the frond growth was basiplastic for the petiole head and acroplastic for the primary pinnae. The metric method is applied to explore the architecture of four living fern species (*Angiopteris evecta* (Forst.) Hoffm., *Matonia pectinata* R.Br., *Sphaeropteris cooperi* (F.Muell.) R.M.Tryon, and *Woodwardia unigemmata* (Makino) Nakai). *Weichselia* architecture results extremely ordered and regular in comparison with the primary pinnae variation of the living species.

## Introduction

*Weichselia reticulata* (C. Stokes et Webb) Fontaine was first described in the Wealden facies of England [1]. Because of its abundance, wide distribution and remarkable architecture, *Weichselia* has received much attention in taxonomy [2,3,4,5], histology [6,7,8], palaeoecology [9,10], and palaeobiogeography, where it has been used as a reference taxon in several Early Cretaceous floristic provinces [11,12,13]. The foliage was related to the stipe *Paradoxopteris* Hirmer [14], and the fertile structures were also described in detail [15,16,17].

The *Weichselia reticulata* frond was first reconstructed as a pedate head with radially arranged bipinnate sterile pinnae by Bommer, based on articulated material from the Wealden facies of an unspecified locality in Belgium [2]. Dabber gave an alternative reconstruction based on specimens from the Barremian of Quedlinburg (Germany), where he interpreted *Weichselia* as a stem bearing radiating primary pinnae [3]. Later, Alvin proposed a whole plant reconstruction of the fern: with an upright stipe bearing several large fronds, each consisting of a petiole ending in a pedate head as Bommer [2] had suggested [4]. Alvin compared such frond architecture with that of *Matonia* R.Br. ex Wall [4]. This reconstruction is generally accepted today, and although some slight modifications have been published [18,19], the general morphology of the frond is maintained.

In this study a significant number of pinna fragments, a nearly whole pinna, and a fairly complete frond of *Weichselia reticulata* are measured and statistically analysed. The specimens, all of them exceptionally preserved, come from Las Hoyas, an upper Barremian fossil lagerstatte located in the La Huérguina Formation (Southwestern Iberia Sector, Cuenca, Spain). We propose a detailed protocol to recompose and locate the position of the fragmentary pinnae along the frond using a set of metric variables. With the use of this protocol, the sample for the analysis is considerably augmented, as it is possible to analyse the fragmentary material sequentially, allowing for a correct comparison along the different zones of the pinnae. By assessing the variability of the variables used and how they correlate with each other, we provide a unique representation of the *Weichselia* architecture, clarifying its growth pattern. The plagiotropic or orthotropic frond position, and the acroplastic, periplastic or basiplastic growth process are discussed. Additionally, the method for architecture representation is tested on five extant fern pinnae of four different species. The extant ferns comprise *Matonia pectinata* R.Br., a member of the Matoniaceae family to which *Weichselia* is usually attributed. Two arborescent ferns belonging to different orders, Marattiales (*Angiopteris evecta* (Forst.) Hoffm.) and Cyatheales (*Sphaeropteris cooperi* (F. Muell) R.M Tryon), together with an herbaceous fern with large fronds (*Woodwardia unigemmata* (Makino) Nakai) have been incorporated for comparison.

## Geological setting

Las Hoyas is located in the south-western Iberian Ranges, near the city of Cuenca, Castilla-La Mancha, Spain. The fossil-bearing beds consist of 280m thick finely laminated limestones [20], which form part of the La Huérguina Formation [21]. Las Hoyas fossil site has been dated late Barremian based on fossil charophytes, ostracods, and palynomorphs [22,23,21]. These limestones were produced in the context of a continental (freshwater) subtropical, seasonal, carbonate wetland that overlay a low-relief karstic terrain [24,20], with no evidence of any marine influence [25,26]. The specimens studied were recovered from several layers from the restricted area of the Las Hoyas locality (S1 Table).

### Studied material

**Fig 1:**
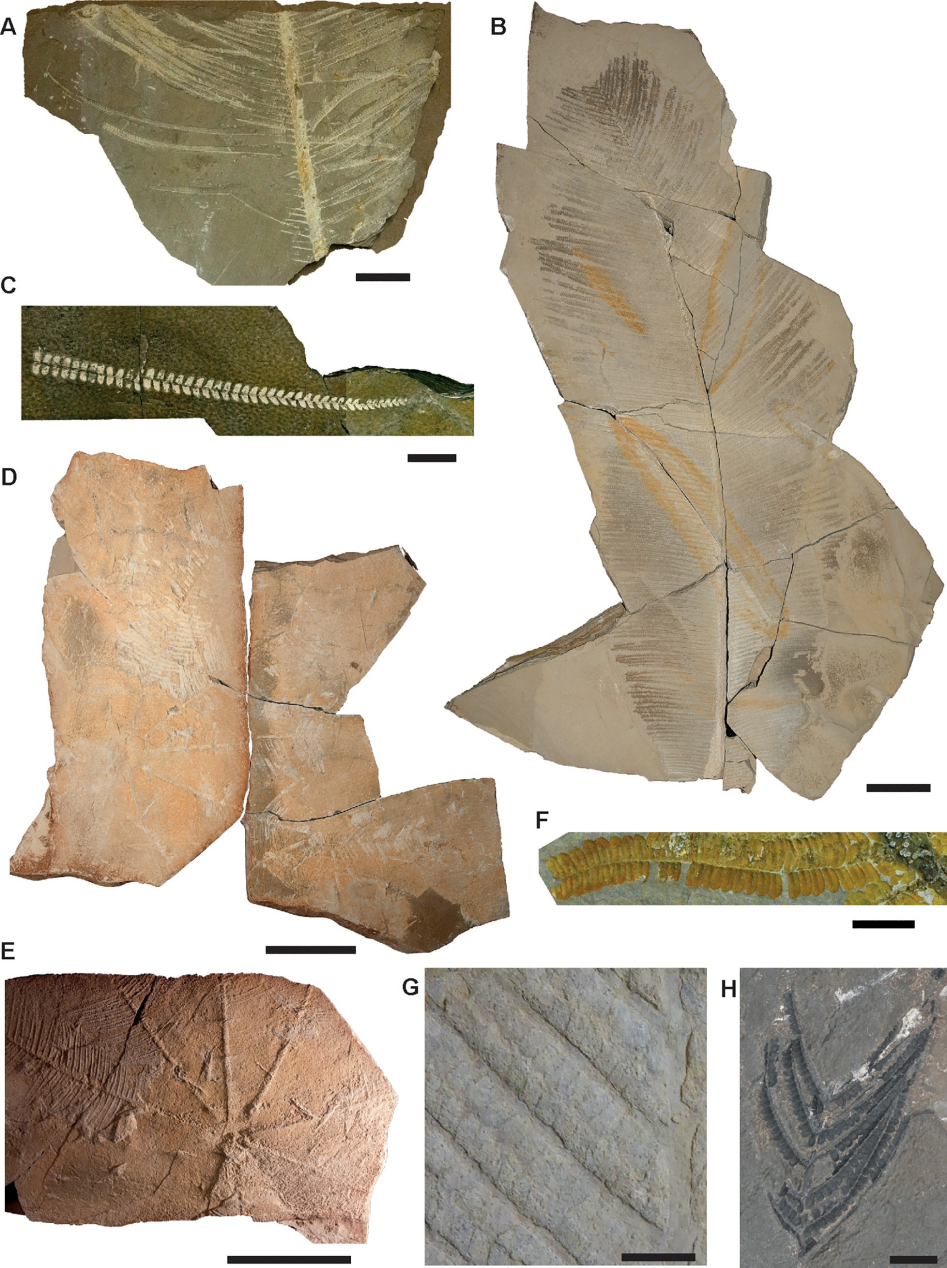
Photographs of sterile pinnae of *Weichselia reticulata* collected from Las Hoyas fossil site. A, large isolated fragment of primary pinna with complete secondary pinna (MCCM-LH 18059B) scale bar = 5cm. B, primary pinna template (MCCM-LH 2859) scale bar = 10cm. C, isolated fragment of secondary pinna (MCCM-LH 16633) scale bar = 1cm. D-E, nearly complete juvenile frond (MCCM-LH 17327), D whole fossil preserved, E detail, scale bars = 10cm. F, detail of basal zone of secondary pinna (MCCM-LH 32554) scale bar = 1cm. G, close-up of a primary pinna impression (MCCM-LH 37758), scale bar = 0.5cm. H, isolated charred fragment of primary pinna (MCCM-LH 30067) scale bar = 1 cm.

Twenty-eight *Weichselia reticulata* specimens from the collection of Las Hoyas (LH) were analysed. They mostly consist of sterile primary and secondary pinnae with a wide range of sizes and levels of completion. This sample includes three relevant specimens: a complete secondary pinna (MCCM-LH18059B), a nearly whole primary pinna (MCCM-LH 2958) lacking part of the base, and a fairly complete frond with the petiole head (MCCM-LH 17327) but lacking some primary pinnae and the apical zone of the pinnae that are preserved (Fig 1). None of them shows sori. The fossils show different types of preservation; most are impressions and charred compressions, and, in some cases, they are covered with iron oxides or calcite (S1 Table). Bearing in mind that previous works based on a very large sample of *Weichselia* fragments from Las Hoyas [27] detected no biases in the measurements due to preservation, all the specimens in this study are considered to be comparable.

The extant material analysed consists of five pinnae of four species: *Matonia pectinata*, *Angiopteris evecta*, *Sphaeropteris cooperi* (primary and secondary pinnae), *Woodwardia unigemmata*. This selection of species aims to explore differences and similarities in frond architecture between distinct groups of ferns and plant habits. *Matonia pectinata* and *Angiopteris evecta* are representatives of two families where *Weichselia* has been previously included, Matoniaceae and Marattiaceae respectively. *Matonia pectinata* is herbaceous, whereas *Angiopteris evecta* is arborescent. *Sphaeropteris cooperi* is a tree fern with a similar habit to the one proposed in the reconstruction included in Poyato-Ariza and Buscalioni [19]. Finally, *Woodwardia unigemmata*, is an herbaceous fern with large leaves, and allows to explore another possible habit for *Weichselia*.

The specimens were photographed with a digital camera Nikon D5100. Measurements were taken on scale-calibrated photographs with Image J 1.49v. [28], or directly on the specimens.

To identify each measurement, the primary pinnae of the same frond, secondary pinnae of the same primary pinna, and the primary and secondary pinnae found of the same specimen, are written down with the specimen number followed by a hyphen and a number (e.g., MCCM-LH 17327–1). When the left and right sides of the pinnae could be measured, they were identified with the letter L, for the left-hand side, and R for the right-hand side. (See S1 Fig. for notation).

Listed material: (1) fossil specimens housed at MCCM, Museo de las Ciencias de Castilla-La Mancha (Cuenca, Spain), LH, Las Hoyas collection: *Weichselia reticulata*: MCCM-LH 2442B, 2549, 2568, 2591, 2788, 2958, 9689A, 13262, 13514, 16633, 17327, 18059A/B, 18423, 27066a, 28114, 29913, 30067, 30215B, 30139B, 31275, 32554, 37030, 37757, 37758, 37759, 37760, 37761, and 37762; A and B correspond to the bottom and top slabs, respectively. (2) Extant specimens housed at Kew Gardens (UK): *Matonia pectinata*, herbarium specimen K001109484; *Angiopteris evecta* and *Woodwardia unigemmata*, live specimens. (3) Extant specimens housed at Jardin botanique de Lyon (France): *Sphaeropteris cooperi*, live specimen.

## Methodological procedure

The study of plant architecture aims to understand the position, distribution and shape of components such as stems and leaves, and involves quantitative analysis or morphometrics [29,30]. Pioneer studies on plant architecture suggested diverse models in tropical angiosperm trees [31]. Further studies were centred on the modelling of living plants in general, and different methods of representation were proposed [32,33, 34]. Most of these models are based on the branching patterns of seed plants [35,36]. Nonetheless, Campbell [33] suggested that they could also be applied to ferns and proposed three methods of representation: (1) Branching Algorithms (BA), (2) Iterated Function Systems (IFS) and (3) Lindenmayer Systems (L-Systems). BA is the simplest and creates plain, rough architecture models of fern leaves. It is a good descriptive method that provides information on the variation pattern throughout the pinna. IFS and L-Systems provide better models of fern leaves than BA, but they are unsuitable for leaf description. Despite these methods, so far, only a few architectural studies on living ferns have been carried out [30,37], and, as far as plant fossils are concerned, they are restricted to the Palaeozoic [29,38,39,40].

### Measurements and ratios

**Fig 2:**
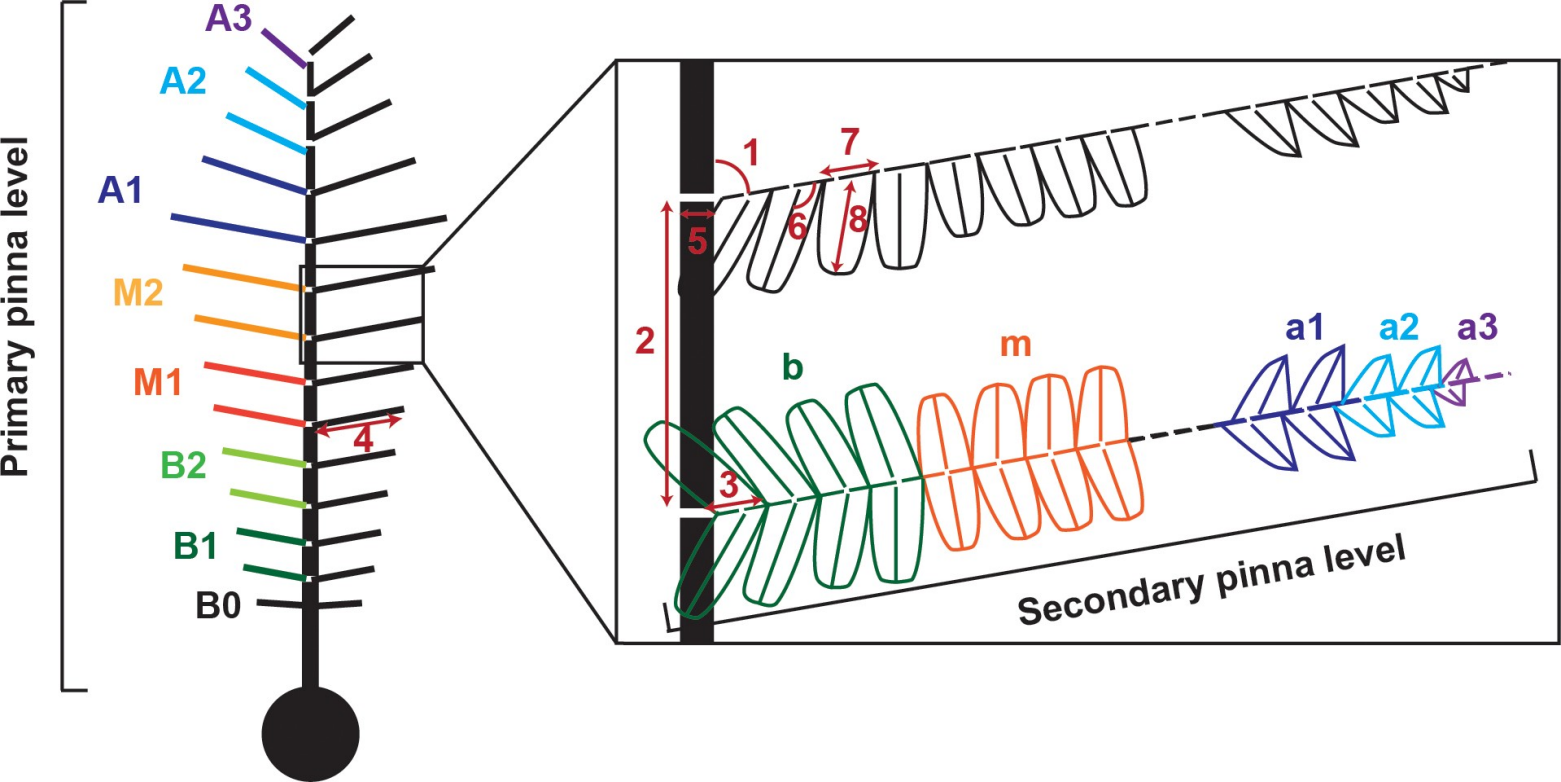
Criteria used for the metric analysis and partition of the pinna in zones. The zones are labelled with the letters B, Basal, M, Middle, and A, Apical. Numbers stand for the measurements taken at the primary pinna level (PP) and the secondary pinna level (SP). (1) IA-PP, secondary pinna insertion angle; (2) DBP-PP, distance between secondary pinnae; (3) FSL, length of the first segment of the secondary pinna; (4) TL-PP, total length of the secondary pinna; (5) RW, width of the primary rachis; (6) IA-SP, pinnule insertion angle; (7) DBP-SP, distance between pinnules; and (8) TL-SP, total length of the pinnule.

To analyse how the secondary pinnae change along the primary pinna in size and disposition, the variables used for the Branching Algorithms and proposed by Campbell [33] were reassessed. Campbell’s variables were: (1) the length of the initial segment of each branch (FSL), (2) the angle at which each branch arises (IA), (3) the interval ratio (IR), and (4) the branching ratio (BR). In this study, the rachis width (RW) and the tapering ratio (TR) are added for the first time. The tapering ratio is calculated from the rachis width to account for the decrease in width along the length of the primary pinna. The criteria used to calculate the measurements and the ratios are presented in Fig 2, and Table 1. The interval ratio and the tapering ratio are calculated numerically, whereas the branching ratio represents the pair x, y value (i.e., first segment length, FSL, and distance between pinnae, DBP).

**Table 1:** Ratio calculations for primary and secondary pinna.

Ratios	Level	Ratio calculation
Tapering ratio (TR)	Primary pinna	RW/previous RW
Interval ratio (IR)	Primary pinna	DBP-PP/previous DBP-PP
	Secondary pinna	DBP-SP/previous DBP-SP
Branching ratio (BR)	Primary pinna	DBP-PP/FSL or DBP/TL-PP
	Secondary pinna	DBP-SP/TL-SP

Some measurements could not be obtained in the ill-preserved parts of some primary and secondary pinnae of the fossil specimens. These measurements are: the rachis width and tapering ratio, which could not be measured for the secondary pinnae, as the actual width of the secondary rachis was largely hidden in some areas by the “butterfly” insertion of pinnules (Fig 2); the pinnule insertion angle in most specimens, which was estimated based on 21 incomplete secondary pinnae with clearly visible mid-veins; and finally, the first segment length was not measured in MCCM-LH 17327. For this latter specimen, in order to calculate the branching ratio, the total length of the secondary pinna (TL-PP) was used, instead of the first segment length (FSL).

### Analysis

The protocol combines data provided by the fragments and the most complete fossil specimens (primary pinna MCCM-LH 2958 and secondary pinna MCCM-LH 18059B-19) that were used as a template. The primary pinna was divided into seven equal segments of 20 secondary pinnae each. They were called *B1*, *B2*, *M1*, *M2*, *A1*, *A2*, and *A3* zones (*B* for basal, *M* for middle, and *A* for apical). The secondary pinnae were divided into three zones, again *b* for basal, *m* for middle, and *a1*-*a2*-*a3* for apical. Every zone corresponds to an abrupt change in the interval ratio (Fig 2).

The measurements and the ratios of the primary pinna MCCM-LH 2958 and the secondary pinna MCCM-LH 18059B-19 were calculated, and once the fragmentary specimens were processed, they were located in their precise position along the templates of the primary and secondary pinnae in accordance with their metrics. To test the precision of the relocation, a Kolmogorov-Smirnov test was used, comparing the values of the template and isolated fragments for each zone.

First, the partition into zones of the primary and secondary pinnae is considered, so that every isolated fragment is placed in its most probable position on the basis of some precise variables. Next, measurements and ratios of the primary pinna are described to show how the secondary pinnae change, incorporating the whole sample already arranged into zones. In order to explore and summarize the metric variability of *W*. *reticulata* specimens from Las Hoyas, a Principal Component Analysis (PCA) [41] is carried out on all data in Fig 2. This PCA is based on a correlation matrix, as both angles and lengths are included in the analyses. Finally, measurements and ratios of the secondary pinna are provided to test the pinnule variation along the secondary pinna. A PCA is unnecessary for the secondary pinnae, as only the distance between pinnules and the total length of pinnules could be measured.

A PCA including the *Weichselia reticulata* template (MCCM-LH 2958) and the extant species is also performed. In this case, the values used (IA, DBP and TL) are standardised to avoid size biases.

## Results

### Isolated fragment relocation

The most plausible location of the isolated *Weichselia* fragments along the whole pinna length is ascertained by the comparison of specific variables with those of the most complete specimens. These specimens are used as templates and are MCCM-LH 2958 for the primary pinnae, and MCCM-LH 18059B-19 for the secondary pinnae.

**Fig 3:**
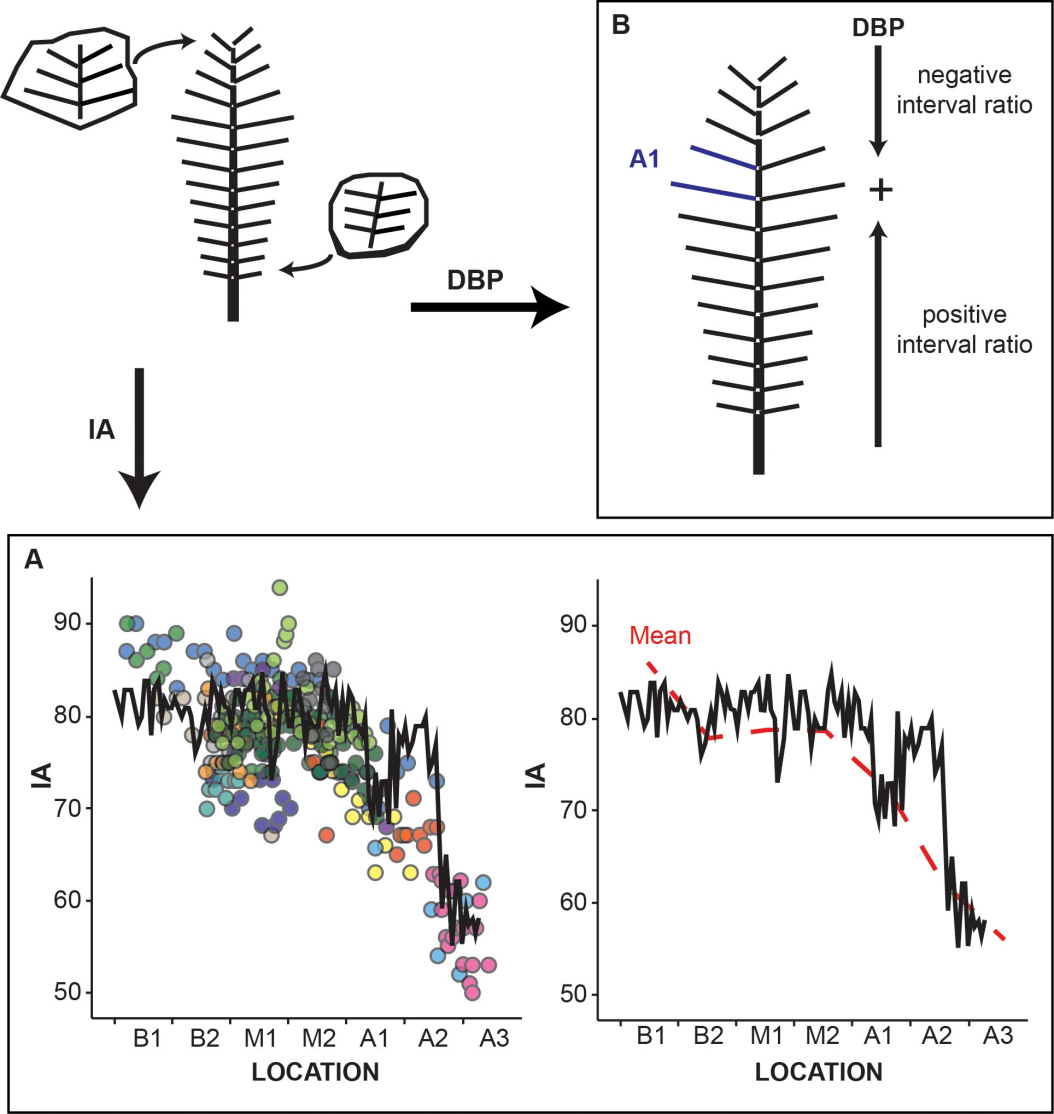
Relocation of the fossil fragments for the primary pinna. The relocation of *Weischelia* fragments is based on: A, the insertion angle (IA), and B, the distance between pinnae (DBP). A, graphs representing the insertion angle (IA) of all the primary pinnae studied. Left plot shows the IA of each secondary pinna; the solid line represents the template (MCCM-LH 2958), and coloured dots represent each specimen. Right plot shows the IA mean (dotted line) of all specimens except for the template, solid line represents the insertion angle of secondary pinnae of the template. IA in degrees. B, depicts the limits for the positive and negative values of DBP along the primary pinna.

In the primary pinna, the results of the insertion angle (IA-PP) and the interval ratio of the template MCCM-LH 2958, show that IA follows a continuous metric with lower values towards the apical part, whereas the interval ratio is positive at the basal zones (*B0-M1*) and negative at the apical zones (*M2-A3*; Fig 3). The pattern provided by both variables allows for the repositioning of the small isolated fragments (e.g., a low insertion angle and a negative interval ratio correspond to an apical zone). The statistical comparison of the distributions of the template and the fragments shows that there are no significant differences between them (see S2 Table for non-parametric test results). Specimens preserving the base of the primary pinna could not be relocated into the zones provided by the template, so an extra zone, *B0*, was added.

To locate the isolated secondary pinnae fragments, the distance between pinnules (DBP-SP) and the total pinnule length (TL-SP) of every isolated fragment is compared to the values obtained for the secondary pinna of MCCM-LH 18059B-19. DBP has a rather steady distribution along the *b-a* zones of the secondary pinna, whereas TL-SP decreases continuously from base to apex. The difference in the total length of the pinnule (TL-SP) along the total length of the secondary pinnae delimits the *b* zone which corresponds to 10% of the secondary pinna, *m* zone corresponding to 65%, and *a* zone that conforms 25%.

### Primary pinna of *Weichselia reticulata*

#### Measurements

**Fig 4:**
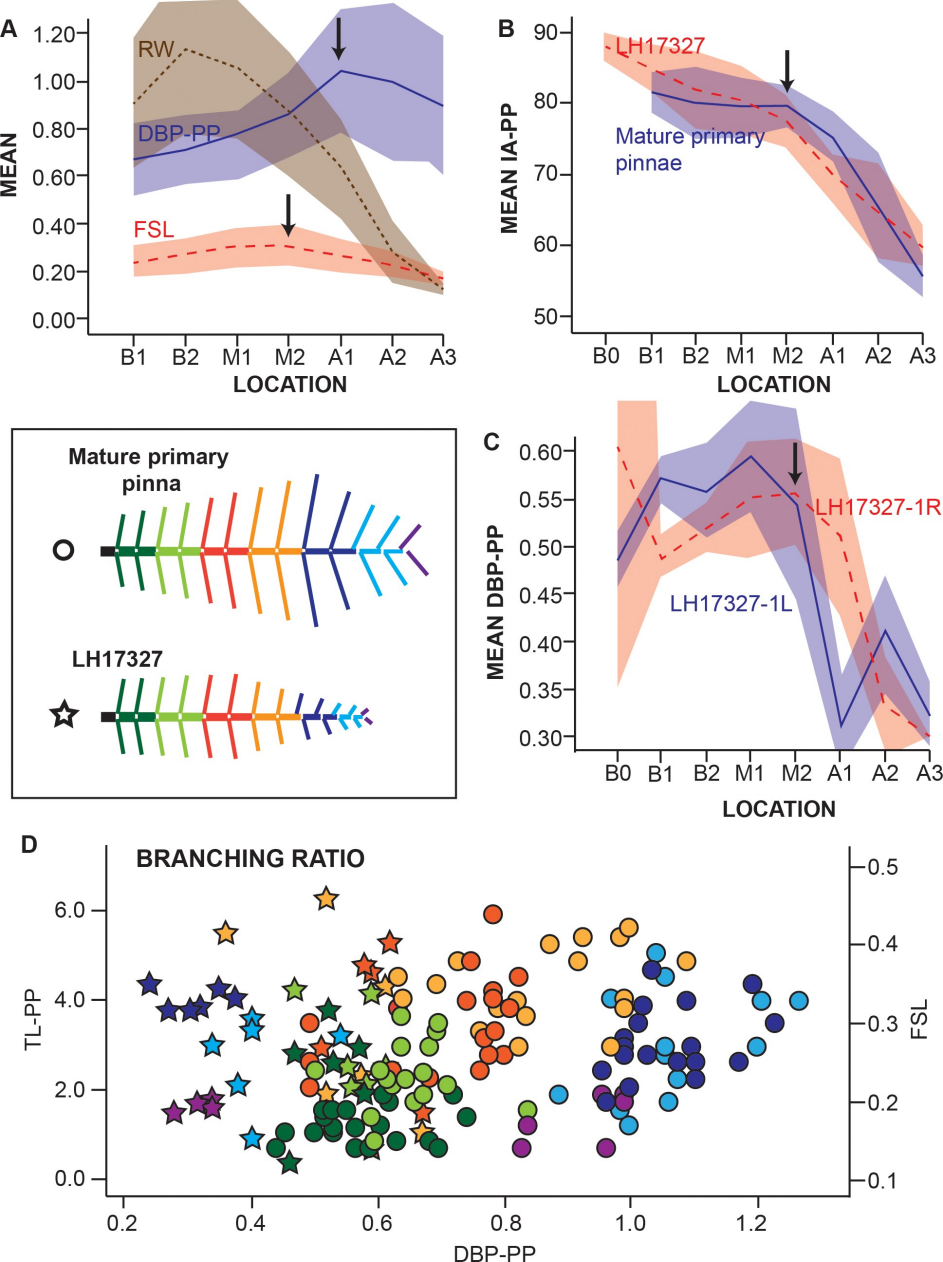
Metrics of the primary pinna of *W*. *reticulata*. A, mean and standard deviation of the measurements rachis width (RW), distance between secondary pinnae (DBP-PP), and first segment length (FSL) for each zone of the primary pinna. B, mean and standard deviation of the insertion angle (IA-PP) of mature pinnae and of specimen MCCM-LH 17327 for each zone of the primary pinna. C, mean distance between secondary pinnae of both sides (left and right) of the primary pinna MCCM-LH 17327–1 for each part of the primary pinna. D, Branching ratio of MCCM-LH 2958 (dots) represented by the pair (x = DBP-PP, y = FSL); and MCCM-LH 17327 (stars), represented by the pair (x = DBP-PP, y = TL-PP). FSL and TL-PP have a strong correlation (Pearson correlation = 0.597, p-value = 0.00). All linear measurements in cms.

Once the isolated fragments have been relocated along the primary pinna, the variability of the four measurements (FSL, DBP-PP, RW and IA-PP, see Fig 2) may be examined (Fig 4A and 4B). The four measurements studied on the primary pinna show different patterns along the defined zones (Base-Middle-Apex). The total length of the secondary pinna (TL-PP) was used only when the length of the first segment of the secondary pinna (FSL) was not preserved. These two measurements have a strong linear correlation (Pearson correlation = 0.597, p-value = 0.00).

The rachis width (RW in Fig 4A) tapers constantly along the primary pinna towards the apical zone (zone *A3*). The broadest rachis width observed occurs in zone *B2*, since only one specimen (MCCM-LH 2958) preserves zone *B1* and the other specimens that preserve *B2* are larger. The standard deviation in *B2* and *M1* reflects a higher variation than the one observed from *M2* to *A3*.

The tendency changes around zone *M2* for the measurements (FSL, DBP and IA; Fig 4A and 4B). The secondary pinna insertion angle (IA-PP) is rather regular from *B1* to *M2* of the primary pinna (Fig 4A and 4B). The length of the first segment of the secondary pinna (FSL) and the distance between secondary pinnae (DBP-PP) tend to increase (Fig 4A) up to *M2*. From the beginning of zone *M2* to *A3* the variables IA-PP and FSL decrease constantly, whereas DBP-PP is unvaried and subsequently decreases at the apical zone. This measurement shows a greater standard deviation between *A1-A3* (Fig 4A).

#### Juvenile specimen

The frond (MCCM-LH 17327) shows lower values for the rachis width and the total length of the secondary pinna. The trajectory of DBP-PP on this specimen is different from the template and isolated fragments (Fig 4C). The distances between the secondary pinnae along the primary decrease very abruptly in *M1*, instead of increasing as occurs in the remaining specimens. However, the insertion angles of the secondary pinnae (IA-PP) are very similar to other specimens, indicating that this variable is conserved irrespective of the number of secondary pinnae or the size of the primary pinna. This characteristic, combined with the shorter distances between secondary pinnae from zone *M2*/*A1* to the apex, and the lower values for the rachis width in MCCM-LH 17327, suggests this frond is a juvenile specimen.

#### Ratios

The study of the tapering, the interval, and the branching ratios shows that all the specimens measured are very similar. However, two of them deviate from the mean values obtained for the rest of the specimens from Las Hoyas, i.e., MCCM-LH 2958 and MCCM-LH 17327 (Fig 4D, S3 Table and S4 Table for tapering and interval ratios).

The tapering ratio is low and unvarying for all the specimens (see S4 Table for values). Significant differences in the template specimen MCCM-LH 2958 occur and two different tapering ratios are observed; width is constant up to zone *M2* and tapers significantly from *M2* to *A3*. Also, the central pinna of the juvenile frond (MCCM-LH 17327–4) shows a particularly low tapering ratio, which differs both from other primary pinnae of the same frond and from the rest of the specimens.

**Fig 5:**
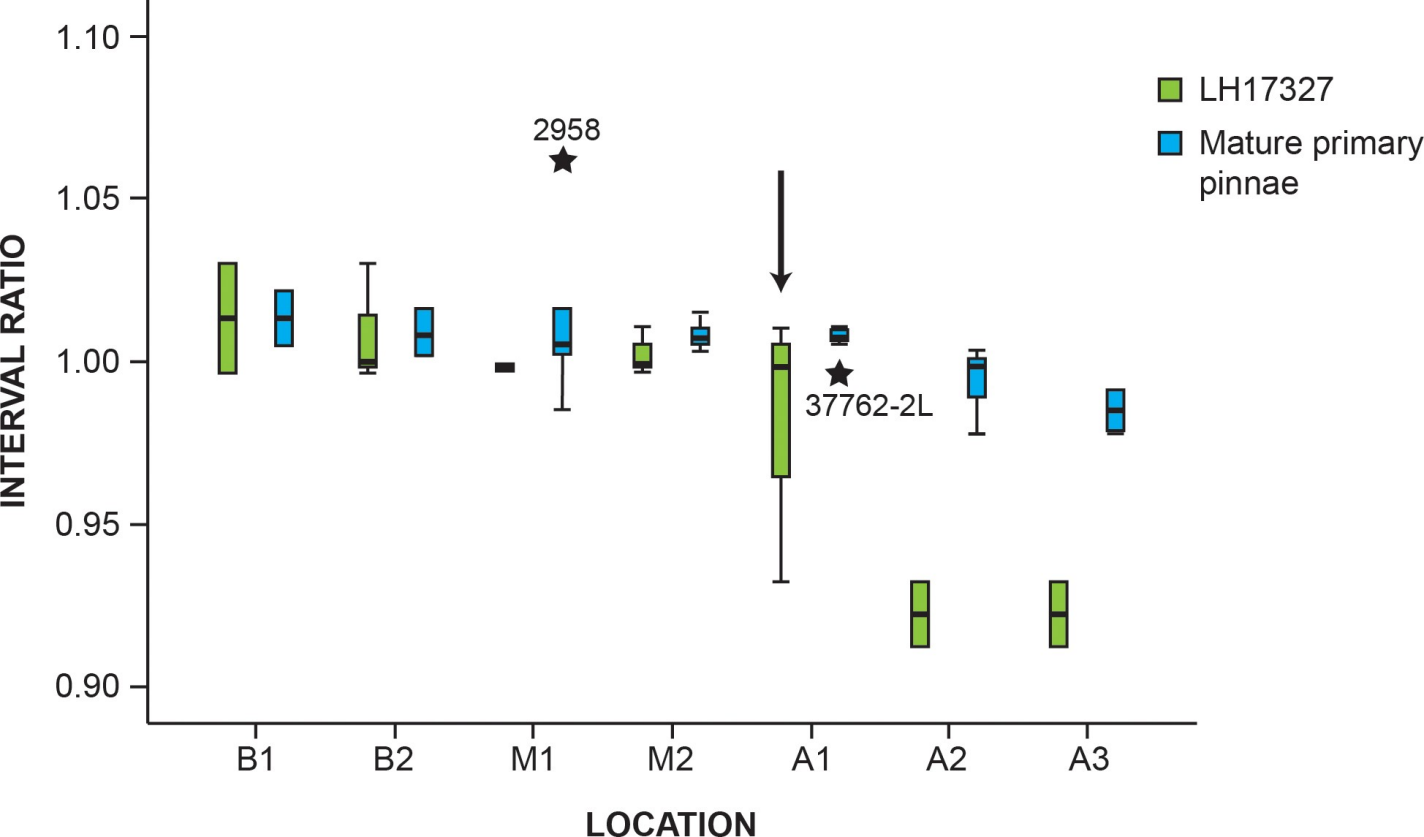
Box and whiskers graph of the interval ratio. Comparison between the mature primary pinnae and the juvenile frond MCCM-LH 17327.

A very high interval ratio from *B1* to *M2* is observed in MCCM-LH 2958, which is different from the lower values obtained for all the primary pinnae in the juvenile frond MCCM-LH 17327 (see S3 Table for values). Strikingly, the variability in the interval ratio among the primary pinnae that constitute the juvenile frond is significantly higher than that of the remaining adult specimens that contain the template frond and the isolated fragments (Fig 5). Left and right sides from the same specimen were measured to explore the symmetry of the interval ratios. The primary pinna is asymmetrical due to the fact that one of the sides has interval ratios around 1, indicating that the distance between secondary pinnae is constant along the primary pinna, and in the counter side interval ratios differ to 1, denoting that the distance between secondary pinnae increases or decreases along the primary pinna. The ratio´s values alternate left to right every 20 to 40 secondary pinnae. The asymmetry can be graphically observed in Fig 4C for MCCM-LH 17327-1L and R. (Fig 4C).

The branching ratio results in an arrangement that follows the serial position (coloured dots in Fig 4D) of the secondary pinnae along the primary one. The graph figured (Fig 4D) includes the juvenile frond (MCCM-LH 17327) and the adult template (MCCM-LH 2958). Both specimens have a similar horseshoe pattern due to serial secondary pinnae, and in both the middle zone is widely variable. However, in the juvenile pinnae the two variables are smaller at the apex, whereas in the adult template the base shows the smallest values for both variables.

#### Principal component analysis

**Fig 6:**
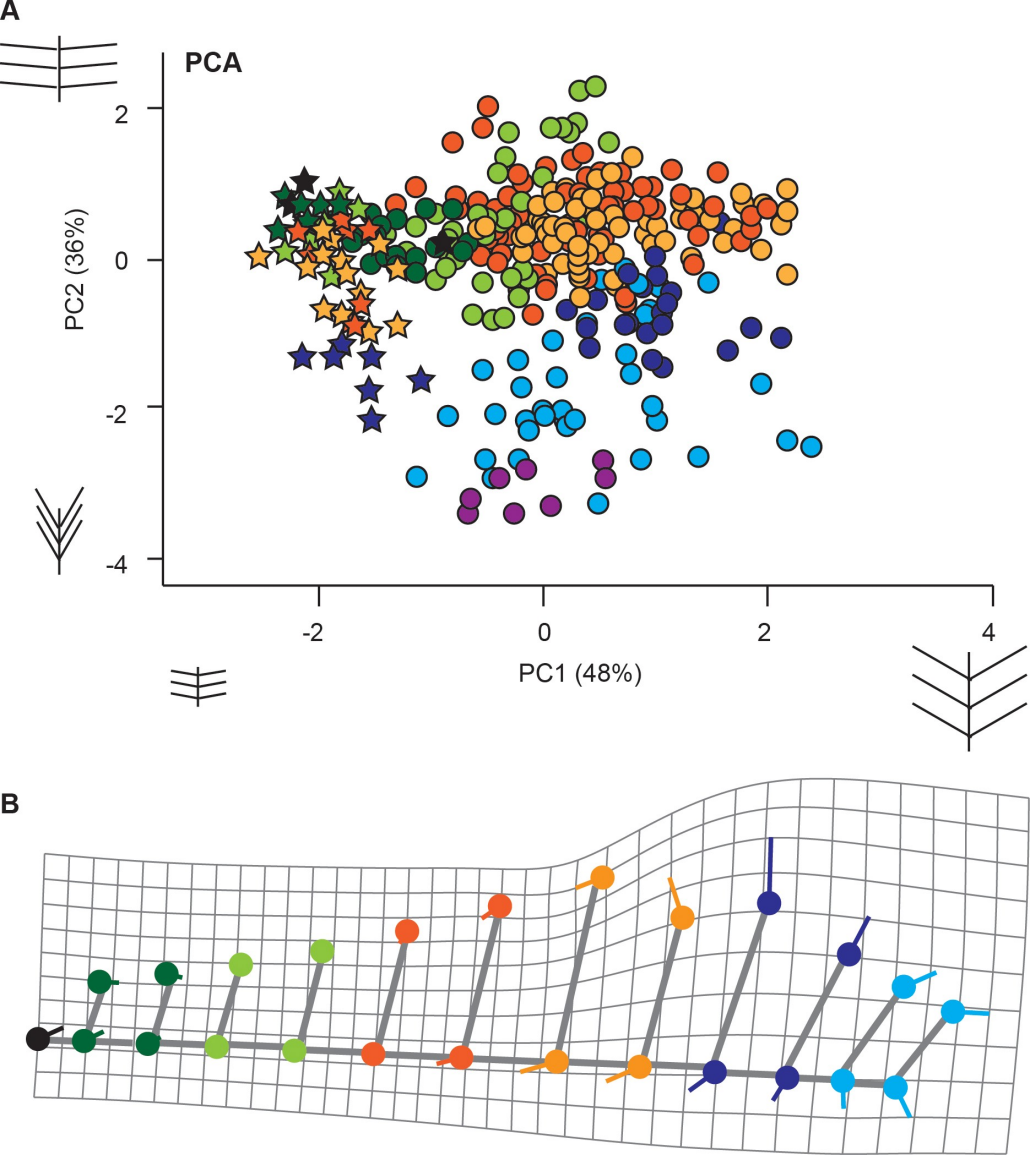
PCA and deformation grid. A, The PCA of the primary pinnae includes the juvenile frond (MCCM-LH 17327, stars in the graphic), the adult template (MCCM-LH 2958), and isolated fragments (dots in the graphic). B, Deformation grid of the superposition of the adult specimen MCCM-LH 2958 over the mean of the primary pinnae of the juvenile frond using MorphoJ. The maximum variation corresponds to zone M. The lateral variation of the rachis observed on the grid is an artefact due to the one on one comparison of only one side of the pinnae. The colour code is the same as in Fig 3.

The PCA was performed without the rachis width to minimize the effect of size (Fig 6A, Table 2), because rachis width is most influenced by size, that is, the length and width of the primary pinna. PC1 explains 48% of the total variance and accounts for the length of the first segment of the secondary pinna (FSL), the distance between secondary pinnae (DBP-PP) (Table 2), and, in a much lesser extent, the secondary pinna insertion angle (IA-PP) (Fig 6A). The juvenile frond (MCCM-LH 17327, star symbols in Fig 6A) expresses the differences related to size and the distance between secondary pinnae, in comparison with the adult pinnae. PC1 captures the serial variation between *B* (negative values) and *M* zones (positive values) in the adult pinnae (Fig 6B).

**Table 2:** PCA loadings for PCA of the primary pinna level excluding the variable RW from the analysis.

	Principal Component	
	1	2
**IA-PP**	-0.133	0.953
**DBP-PP**	0.868	-0.210
**FSL**	0.818	0.378

On the other hand, PC2 explains 36% of the variance and captures the serial transformation towards the apical zone. PC2 combines the secondary pinna insertion angle (IA-PP) (with the greatest loading) and the length of the first segment of the secondary pinna (FSL) (Table 2).

### Secondary pinna of Weichselia reticulata

#### Measurements

**Fig 7:**
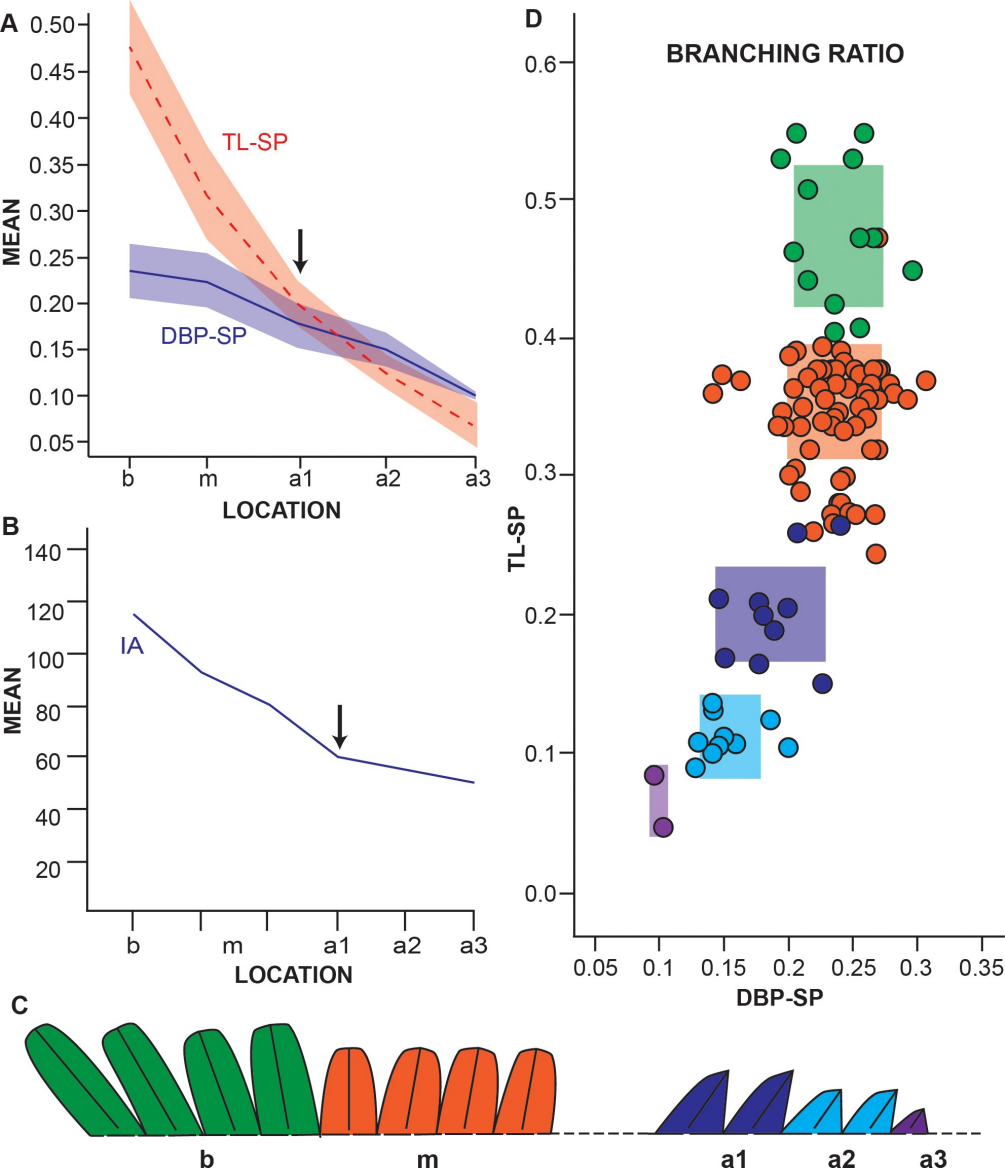
Metrics of the secondary pinna of *W*. *reticulata*. A, mean and standard deviation of the total length of the pinnule (TL-SP) and the distance between pinnules (DBP-SP) at each zone of the secondary pinna. B, mean pinnule insertion angle for each zone of the secondary pinna. C, schematic drawing of the change in pinnule morphology and size throughout the secondary pinna. D, branching ratio of specimen MCCM-LH 18059–19, mean and standard deviation for each zone of the secondary pinna are marked as coloured zones in the graph. All linear measurements in cm.

The measurements of the secondary pinnae attached to rachis and of the isolated fragments show the total length of the pinnules (TL-SP) is considerably longer in zone *b*, subequal in *m*, and diminishing sequentially, in what appear to be three distinct intervals, in *a* (Fig 7A, 7C and 7D). The distance between pinnules (DBP-SP) has a gentle slope decreasing throughout the secondary pinna (Fig 7A), which makes the pinnules wider than they are long in zones *a2* and *a3*.

The pinnule insertion angle (IA-SP) is obtuse in the basal pinnules, from the 1st to 5th pinnule (130°-100°), and diminishes abruptly thereafter to 95°-90°. The middle part of the pinna is at a constant angle of 85°-75°, and finally it decreases abruptly to approximately 60°-50° (Fig 7B). The change in morphology and size of the pinnules is associated with measurement variations related to each zone (Fig 7C).

#### Ratios

The secondary pinnae interval ratio is approximately 1, higher in *b* and *m*, and less than 1 in *a* (see S5 Table for values). In some specimens (MCCM-LH 18059–19, MCCM-LH 2958–127, and MCCM-LH 2568) the interval ratios differ greatly from the other specimens. The asymmetry of the secondary pinnae is random, unlike that of the primary pinnae.

The distribution of the branching ratio values for the secondary pinnae results in four discrete zones (Fig 7D), which clearly differ from the continuous horseshoe pattern of the primary pinnae (Fig 4D).

### Extant ferns

**Fig 8:**
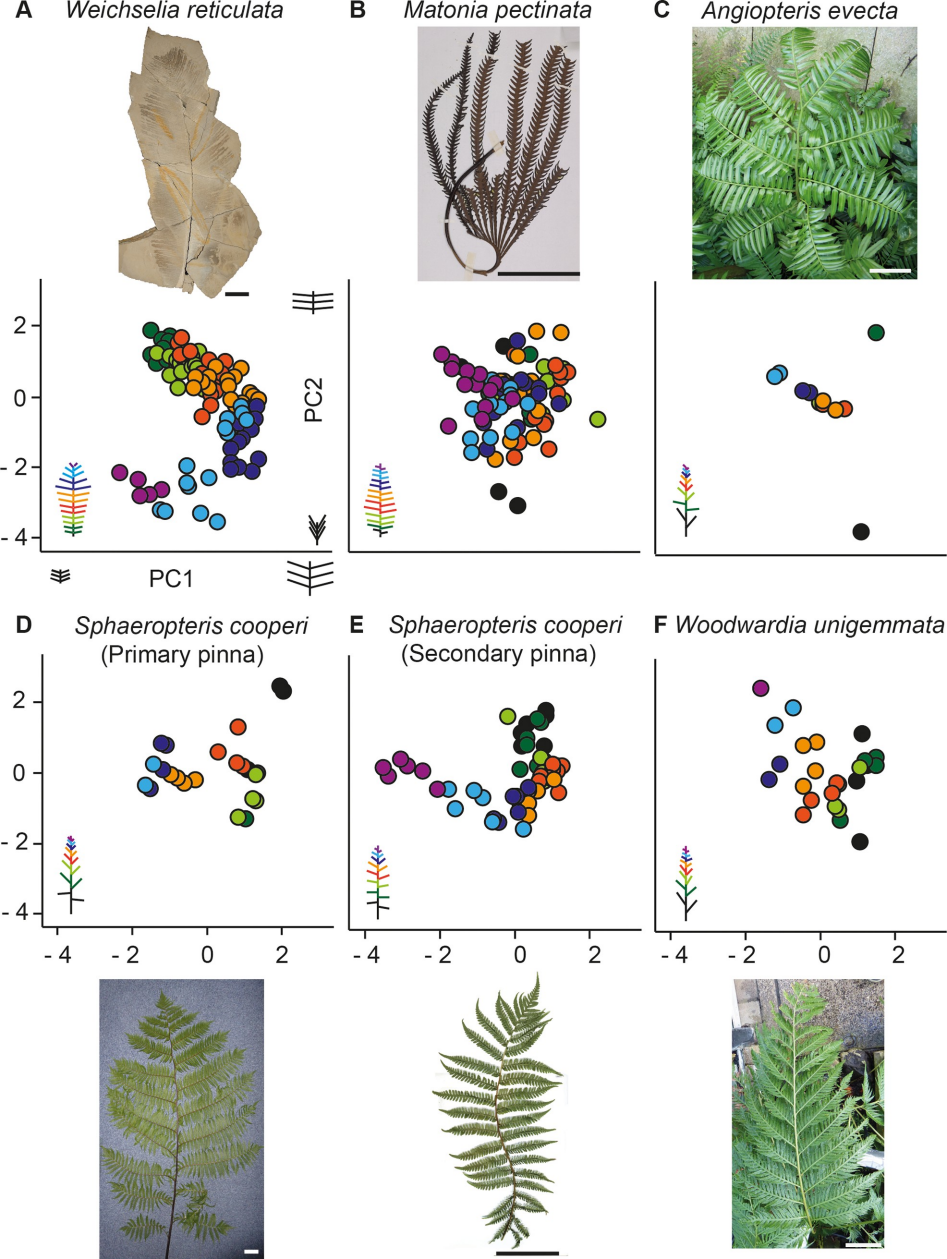
PCA of *Weichselia* and of extant fern pinnae. A. *Weichselia reticulata*. B. *Matonia pectinta*. C. *Angiopteris evecta*. D-E. *Sphaeropteris cooperi* primary and secondary pinnae respectively. F. *Woodwardia unigemmata*. Scale bars = 1cm.

The graphic representation of the PCA includes all the variables except for the RW variable (see S6 Table for loadings). *Weichselia* primary pinna is represented by the template MCCM-LH 2958. All the pinnae (Fig 8A–8F), show in general a sequential variation in the secondary pinnae length (TL) and in the distance between pinnae (DBP) along the primary pinna. This general variation is represented in the PC1 (x-axis). *W*. *reticulata* and *M*. *pectinata* secondary pinnae are shorter (TL) and more packed (DBP) at the base and apex, and longer and further apart in the middle area; whereas in the rest of the specimens the secondary pinnae decrease in size from base to apex (Fig 8). Only *Weichselia* and *S*. *cooperi* clearly depict a parabolic "horseshoe distribution" due to sequential variation in both PC1 and PC2. The PC2 (y axis) is mostly characterized by the insertion angle (IA). The sequential variation is apparent along the PC1 in the basal and middle area of the fossil fern; and along the PC2 in the apical area with a tendency to smaller angles (Fig 8A). In *S*. *cooperi* the parabolic distribution is explained by the insertion angles (PC2) in the secondary pinnae of basal and middle areas; and PC1 in the apical area, where pinnae are smaller and more packed (Fig 8E). PC2 denotes that *Matonia pectinata*, *Angiopteris evecta*, and the primary pinna of *Sphaeropteris cooperi* bear undifferentiated basal and apical areas, with subequal angles and uneven basalmost secondary pinnae. The ordered distribution of *Weichselia* has to be emphasized, as well as the extremely low negative values of PC2 indicating the acuteness of the insertion angle of the secondary pinnae in the apical zone. Strikingly, *Woodwardia unigemmata*, (Fig 8F) has the opposite ordination for PC2, from acute basal secondary pinnae to orthogonal at the apex.

## Discussion

The adequacy of the sample, combining numerous isolated fragments with almost complete specimens, allow to explore the total range of variation of the *Weichselia* metrics. The variables used to relocate the isolated fragments within the pinna are congruent with the measurements and ratios used to describe the primary and secondary pinnae. The results on both fossil and extant ferns corroborate the usefulness of Campbell’s measurements in fern leaf description [33]. Rachis width, however, is notably influenced by size, which is why it is not an accurate enough proxy to explore the zonal differences. Despite the large dispersion of mean values for the ratios in *Weichselia*, the combination with measurements made the ratios reliable [42].

Our quantitative approach to the pinnae variation reveals some relevant aspects on the architectural and growth patterns of *W*. *reticulata*, which shows differences with the previous reconstructions of the fronds of this fern [2,3,4,18,19] (S1 Text) Furthermore, the comparison between the complete small frond (MCCM-LH 17327) and the large specimens allows to recognise the growth types of the frond of this fossil fern. The analysis on extant pinnae shows that different variable combinations conform the architecture of fern pinnae.

### Growth pattern

It is unknown whether *W*. *reticulata* had a periplastic, acroplastic or basiplastic growth. However, our results suggest that the growth pattern must have been acroplastic for the primary pinna (i.e., it grows from base to apex), and basiplastic for the petiole head (i.e., it grows from apex to base). Arguably, the differences observed between the smallest specimen and the remaining specimens substantiate the interpretation of a juvenile frond and an acroplastic growth. The basal zone of the primary pinnae in the juvenile frond is already mature and shows similar values to that of the remaining specimens for all the variables used. Even though the basal part was already fully grown, lower values of the tapering ratio suggest that the rachis still had to grow in width in the apical area. Furthermore, in the middle and apical zones of the juvenile frond, both the low values in the interval and branching ratios (Fig 4D and S3 Table) and the shorter distances measured, substantiate an acroplastic growth. As the primary pinnae grew to an adult condition, the distance between secondary pinnae would have become progressively longer at the middle and apex, up to reaching the values of the adult specimens for all the variables. In fact, living ferns with elliptic shapes similar to *W*. *reticulata* show an acroplastic growth [43] with similar patterns for the branching ratio [33].

**Fig 9:**
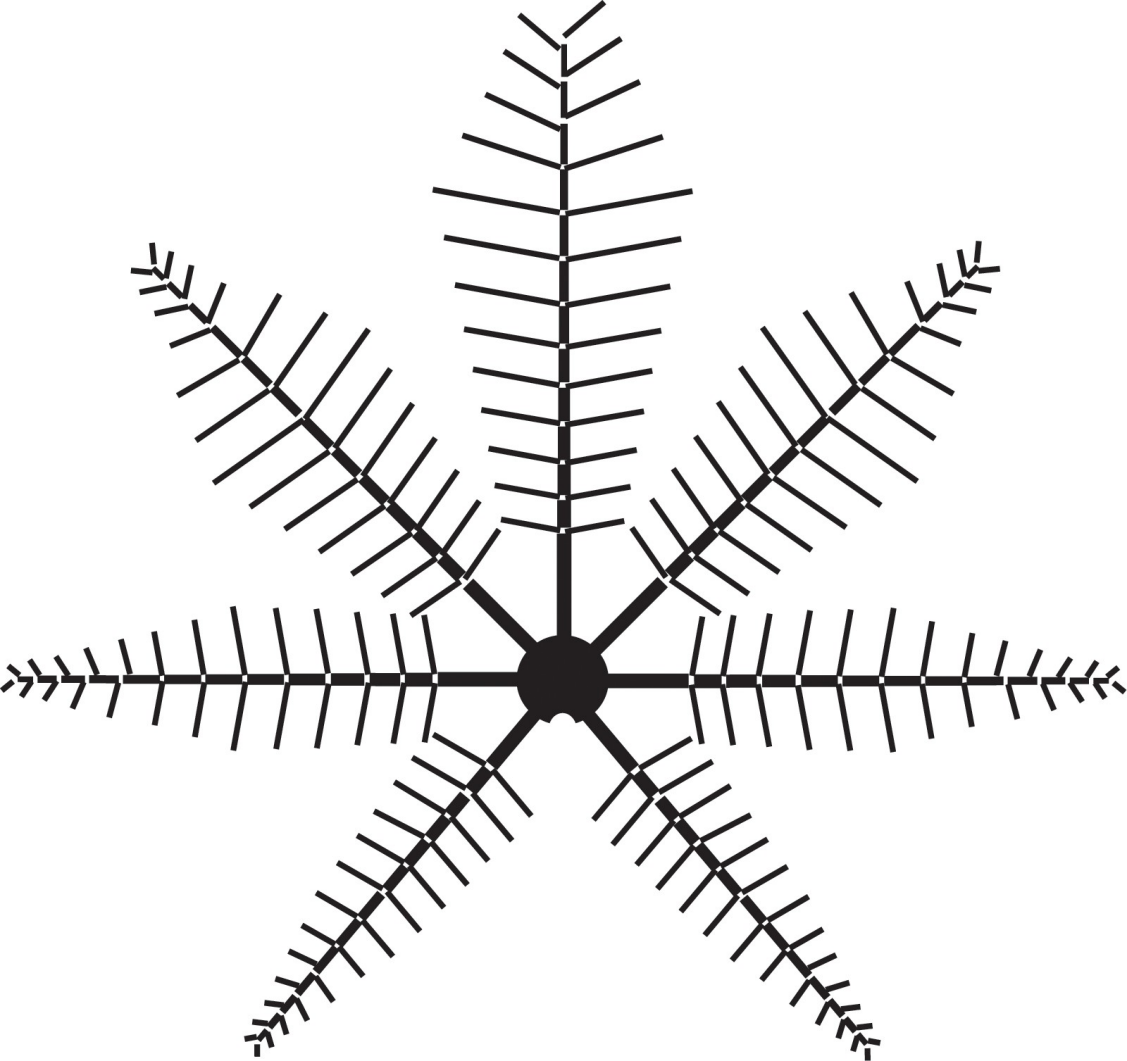
Schematic drawing of the basiplastic growth in the frond of *W*. *reticulata*.

The basiplastic growth of the petiole head is supported by the differences in stage of maturity between primary pinnae observed in the juvenile MCCM-LH 17327. The differences in the interval ratio between primary pinnae of the frond indicate that MCCM-LH 17327–4 would be the most mature, because the interval ratio diminishes closer to the apex than other pinnae (Fig 9). MCCM-LH 17327–4 corresponds to the central pinna as it is inserted opposite to the sulcus of the petiole head. Therefore, the petiole head of *W*. *reticulata* would have had a basiplastic growth.

This type of development, basiplastic for the petiole head and acroplastic for the primary pinnae, also occurs in living ferns with pedate fronds [43,44]. In this case, the architecture of the pedate frond is the result of a modification of a pinnate blade, with a central pinna that continues from the petiole [45]. *Matonia* is a good example of the latter; the central pinna matures first, while the pinnae at the extremes of the pedate structure are the last to mature [44,46]. Although pedate fronds are not exclusive to the family Matoniaceae, they also occur in the Polypodiaceae or the Pteridaceae [3], the pedate structure of their fronds is formed by different strategies, like, for instance, *Adiantum pedatum* L. (Pteridaceae), where the pedate architecture of the frond is the result of a consecutive series of dichotomies [45].

Although fern leaves can exhibit sizes and shapes that differ from their normal morphologies under environmental stress [47], these anomalies produce irregular morphologies. The juvenile frond does not show the irregularities observed in other specimens of the sample, such as MCCM-LH 2958. In that case an anomalous tapering ratio coincides with an exceptionally high interval ratio, suggesting a change in the growth pattern that favours the elongation of the primary pinna. This type of anomaly occurs in living ferns under stress conditions such as lack of light [47,48].

### Architecture

*Weichselia reticulata* has a unique pattern in the sequential variation of the secondary pinnae along the primary pinna which results in a more orderly distribution in comparison with the selected extant species. Neither the large primary pinnae arising from an erected stem (the arborescent ferns *Sphaeropteris cooperi* and *Angiopteris evecta*) nor *Matonia*, share such a regular and ordered distribution of secondary pinnae. Although *Weichselia* and *Matonia* are pedate ferns and develop small and packed basal and apical secondary pinnae, the obtained results do not back any other similarities based on the metric variables. The pedate frond of *Matonia* presents small and simple pinnae that are only once pinnate and the insertion angles are extremely variable in comparison with *Weichselia*. Hence, the shared commonality of small and packed basal secondary pinnae might be understood as an analogy of pedate fronds to prevent overlapping among the bases of the radiating primary pinnae. The regularity observed in the primary pinna of *Weichselia* and the secondary pinna of *Sphaeropteris cooperi* might be related to a controlled growth pattern due to biomechanics, as size and shape affect the stability of leaves [49].

**Fig 10:**
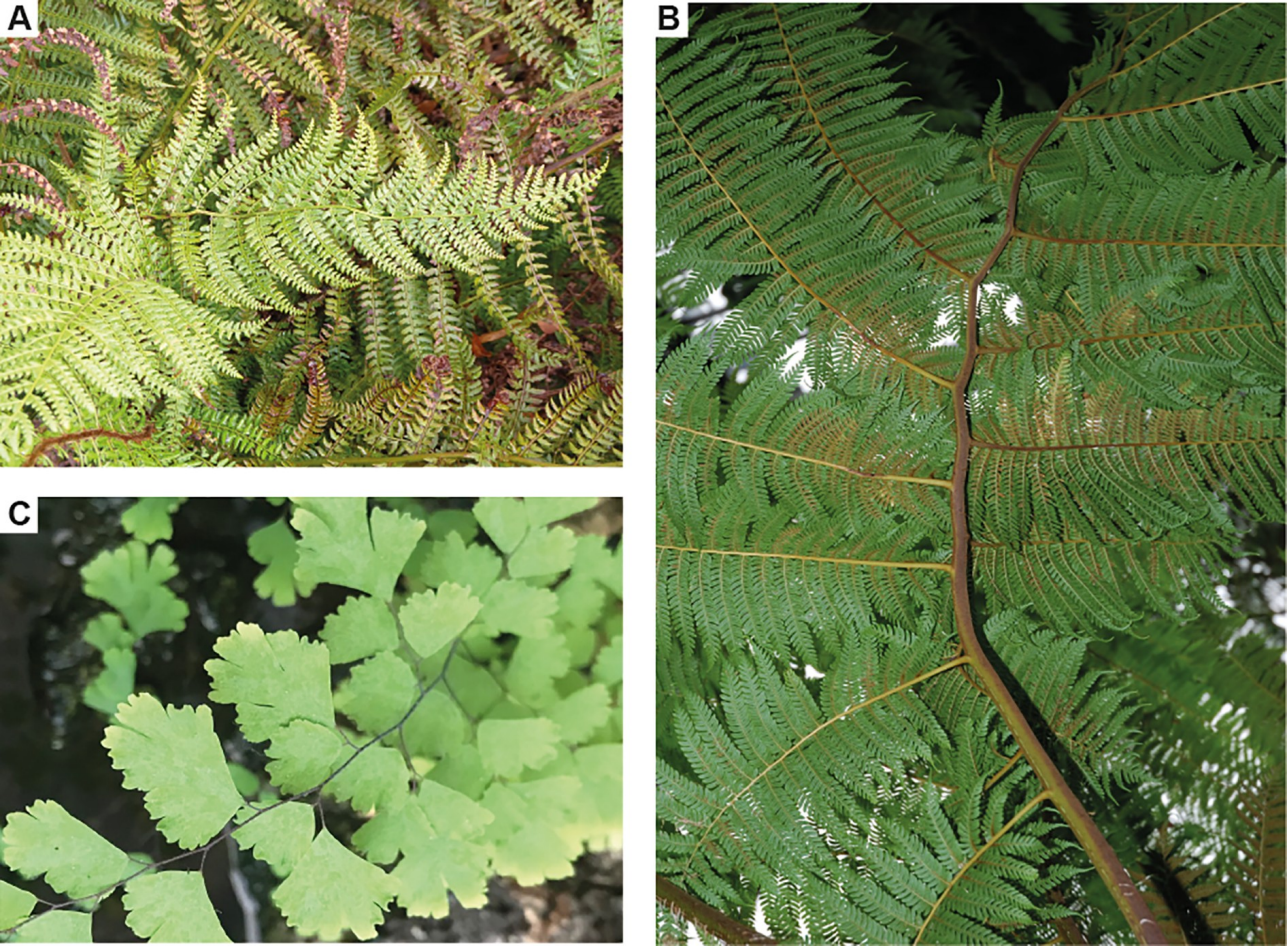
Sinuous rachis in extant ferns. A, S morphology in *Polistichum setiferum* (Forssk.) Moore ex Woynar. B, Sinuous rachis in *Sphaeropteris cooperi* (F. Muell) R.M Tryon. C, Plagiotropic *Adiantum* L. with sinuous rachis.

The measured variables also contribute to highlight some aspects of *Weichselia reticulata* architecture. The umbrella-like frond is not radially symmetrical due to the different growth stages observed in the primary pinnae (Fig 9), showing, in that sense, a general morphology similar to that of the *Matonia* frond. Primary pinnae are elliptical, caused by the differences in the insertion angle of the secondary pinna (IA-PP) along the primary pinna and by the total length of the secondary pinnae (mainly represented by the length of the first segment of the secondary pinna, FSL). Additionally, a plagiotropic position of the fronds can be suggested by the zigzag morphology of the rachis, which is inferred by the alternating unequal growth on each side of the primary pinnae. The different interval ratios on each side of the primary pinnae entail that one side grows while the other remains static, which produces the typical “S” morphologies that can be observed in the rachises of Recent ferns (Fig 10A). Given that such alternating interval ratios are remarkably constant in *W*. *reticulata*, the rachis of this fern could present a regular sinuous morphology like the rachis of some living tree ferns (Fig 10B) and herbaceous ferns such as some *Adiantum* L. species with pinnae in a plagiotropic position (Fig 10C). A sinuous rachis could help to stabilize and distribute the weight of the pinna, increasing the width/length ratio. Also, the alternating unequal growth could cause the change in disposition of secondary pinnae from opposite to subopposite at the basal and middle part of the primary pinna. Precisely, at the apical part of the primary pinna of *W*. *reticulata*, secondary pinnae are alternate. This changing disposition, from opposite to alternate, is general and abrupt as observed by the difference in the distance between secondary pinnae that exists in zone A1 for the left and right sides (Fig 4C). On the contrary, the change in disposition of the pinnules from subopposite to alternate on the secondary pinna is continuous.

The morphology and position of the *Weichselia reticulata* frond here suggested imply that its whole surface would have been exposed to the sun’s irradiance. Nonetheless, some traits, such as the butterfly disposition of the pinnules [50], a resistant cuticle, sunken stomata, and axes with a thick sclerotic outer layer [51] suggest that pinnules would have been protected from the direct sunlight. These traits, have been previously interpreted to be xerophytic, indicating that *Weichselia reticulata* was adapted to extreme drought conditions, and that the occurrence of the fern in subaqueous depositional environments would be due to transport via flooding [7]; on the contrary, Silantieva and Krassilov proposed these traits as a proxy of mangrove-like vegetation [17]. Further contributions on the taphonomy of *Weichselia* will be decisive to solve this dilemma.

## Conclusions

This study revisits the architecture of the sterile fronds of the fern *W*. *reticulata* by measuring the different parts of the frond as a novel approach, which has permitted a detailed description that will be very useful for more accurate reconstructions of this plant in the future. The variables (measurements and ratios) reformulated from Campbell’s contribution [33] provide data to elaborate and hypothesize on the growth pattern of this fern for the first time. The acroplastic growth for the primary pinna and basiplastic growth for the petiole head strengthens the argument of the Matoniaceous affinity of the sterile fronds of *Weichselia*. However, the results of the comparison between *Matonia pectinata* and *W*. *reticulata* suggest this growth similarity might be an analogy, considering the discrepancy on the metric variation of secondary pinnae between these species. Both tree fern fronds and herbaceous fern fronds also show different architectures that differ to *Weichselia*. Further analysis of other organs of *W*. *reticulata* must be conducted in order to shed light on the taxonomic affinities and habit of this fern.

The contribution of the protocol designed to relocate isolated fragments opens a new window to the architectural study of large fossil fronds that are generally found fragmented in the fossil record. Locating fragments in the sequence of the pinna permits the morphometric study of fossil material, allowing for the equivalent comparison with extant or other fossil material, and for more accurate descriptions and reconstructions. Additionally, the reformulated Branching Algorithms method provides a simple approach to the description and comparison of frond architectures of both fossil and extant plants that can even provide insight on the frond growth.

## Supporting information

S1 Text Discussion of the architecture of the frond of *Weichselia* with previous reconstructions. (DOCX)

S1 Fig Notation for the identification of pinnae from the same frond, secondary pinnae of the same primary pinna, and primary and secondary pinnae found of the same specimen. Specimen number is written down followed by a hyphen and a number (e.g., MCCM-LH 17327–1). When the left and right sides of the pinnae could be measured, they were written with the letter L, for the left hand side, and R for the right hand side.
(EPS)

S1 Table Preservation and provenance of each of the specimens studied. Provenance is indicated as the square in the locality where each specimen comes from, and not all specimens have this information.
(XLSX)

S2 Table Statistical significance of the relocation of the isolated fragments for the primary pinna. Calculated by a Kolmogorov-Smirnov Test comparing the distribution of the insertion angle of the secondary pinna (IA) of the template and the relocated fragments.
(XLSX)

S3 Table Primary pinna level interval ratio level and errors for the most complete specimens. Location is coded as shown in Fig 2. When the change in ratio does not coincide with a specific zone, the number of the secondary pinna is used. Location for specimen MCCM-LH 37030 is unknown.
(XLSX)

S4 Table Tapering ratio and errors for the most complete specimens. (XLSX)

S5 Table Interval ratios and coefficients of determination for secondary pinnae with more than 10 pinnules preserved. Location is coded as shown in Fig 2, subzones within zone *a* are not included, T = total pinna. Zones b and m are grouped as DBP is very similar for these zones.
(XLSX)

S6 Table PCA loadings of extant fern analysis. (XLSX)
