# Supplementary material for: A novel approach for the metric analysis of fern fronds: Growth and architecture of the Mesozoic fern Weichselia reticulata in the light of modern ferns
Source: PLoS One. 2019 Jun 27;14(6):e0219192. doi: 10.1371/journal.pone.0219192 (PMC6597107; doi:10.1371/journal.pone.0219192)
Supplement: S1 Text — (DOCX) [file pone.0219192.s001.docx]

Supplementary: Comparison with previous restorations of *Weichselia reticulata*

Whole plant reconstructions in fossil species are not very common, bearing in mind the fact that most of the fossil plants are found as disarticulated plant organs and finding them anatomically connected is very rare (Taylor et al., 2009). Regarding *Weichselia reticulata*, the whole plant has been successfully identified from material from the locality of Bernissart and was partially reconstructed by Bommer (1910). Daber (1968) proposed another reconstruction, based on remains consisting of pedate petiole heads found in Quedlinburg (Barremian, Germany), which were interpreted as stems with a crown of radiating pinnae. Alvin (1971), based on the specimens Bommer (1910) had studied, refuted this reconstruction, and suggested a more detailed reconstruction which is the most accepted to date:

Erect stem, measuring 5-8cm in diameter with an irregular appearance due to the bases of the leaf petioles that are quite close together and spirally disposed, and also to organs directed in the opposite direction of these petioles that have been described as possible bases of rooting organs, similar to the rhizophores present in *Selaginella*. The stem also shows small tubercles that could be modified hairs or ramenta. There are also scars that may represent the presence of aerophores. The vegetative frond is extremely variable in size, especially the petioles, which measure from 0.2cm up to 5cm. Petioles are inserted at a 30° angle to the stem and bear radially disposed primary pinnae at the end, inserted in a pedate head. The flattened distal face of this head is inserted at different angles depending on the size of the petiole. The biggest remains are abaxially orientated forming a 120° with the petiole, whereas the smallest form a 90° angle. This pedate head is divided into 8 to 15 segments that correspond with the bases of the primary pinnae, these vary in size and are not symmetrically disposed. There is an axial sulcus, and the pinnae bases that are closest to it are broader and possibly subdivide. The primary pinnae are 0.2cm to 2.3cm broad and bear secondary pinnae that are inserted closer to the adaxial side of the primary rachis. The distance between secondary pinnae along the primary rachis is from 5mm to 7mm. Inserted opposite to each other in the adaxial side of secondary pinnae are pinnules, orientated in a butterfly disposition. The distance between the midveins of the pinnules is from 1.5mm to 3mm. They vary greatly in size (3mm - 6mm long x 1.3mm – 2.5mm broad) and shape, and have reticulate venation, with 4 to 5 vein meshes between the midvein and margin. The fertile frond is separate from the vegetative frond and has a stout petiole with long primary pinnae that radiate from the petiole head. This primary pinnae bear secondary curved pinnae that tend to join together at the ends, forming a small enclosed space between them where the ultimate rachises are inserted, each with two rows of soral clusters. These “ultimate rachises” would be equivalent to the pinnules in the fertile frond.

Recently, Sender et al. (2015) published a new reconstruction of this species, based on a fertile pedate head, with primary and secondary pinnae attached, from the Albian of Teruel. They represent a vegetative frond at the apical end of the stipe and a fertile frond in a lateral ramification, based on the mention of a possible stem dichotomy by Alvin (1971). The description of fertile structures is also different, the ultimate rachises are not regarded as modified pinnules, but as a third order of pinnae. This implies that, in this species, the vegetative frond would be bipinnate while the fertile frond would be tripinnate.

Additionally, Poyato-Ariza and Buscalioni (2016) suggested another reconstruction, based on material from Las Hoyas. The general appearance of the plant is that of a Recent tree fern, with a wide, tall, erect stem with a crown of pedate leaves at the top. Fronds are represented as marcescent, as it occurs in tree ferns today. The fertile part

The reconstructions of *W. reticulata* (Bommer, 1911; Daber, 1968; Alvin, 1971; Sender et al., 2015; Poyato-Ariza & Buscalioni, 2016; Fig. 4) are not comparable to the study here performed. The frond reconstructions are based on isolated measurements and there is no study of the relationship between parts. These studies provide intervals of measurements, but their location along the different parts of the frond, and relationships between them are lost. Isolated comments on changes in size or morphology along the primary or secondary pinnae are sometimes provided, however, the pattern of variation of the different variables and the relationship between them is essential to understand the architecture of the frond. Although the illustrations provided in these studies are artistic, if both these representations and the comments provided by the authors are analysed together, the reconstructions can be discussed in the light of the information obtained from the architectural study of the frond.

Regarding the general frond architecture, all the reconstructions with pedate fronds (Alvin, 1971; Sender et al., 2015; Poyato-Ariza & Buscalioni, 2016) except for the one proposed by Bommer (1911) show differences in size, or maturity, in the primary pinnae radiating from the petiole head. The pinnae are smaller, or younger, at the adaxial side of the petiole. This is congruent with the differences between primary pinnae observed in specimen MCCM-LH 17327.

Focusing on the primary pinnae, insertion angles of secondary pinnae and their variation along the primary pinna are very different in each reconstruction and are only similar to the architecture described in this study in the reconstruction proposed by Daber (1968), where the angles are 90° for three quarters of the primary pinna, and progressively more acute towards the apex for the last quarter. In this study, instead of 90° the angles were measured to be around 78°-80° for the first three quarters of the primary pinna. All the reconstructions represent secondary pinnae at the same distance of each other along the whole primary pinna, interval ratios are 0, and although Alvin (1971) specifies the distance is variable (0.5-0.7cm), there is no information on the distribution of these measurements along the primary pinna. The first pinnule is always adjacent to the primary rachis all reconstructions except for Bommer (1910), and the first pinnule is the same size along the primary pinna. However, in the study performed, differences in the interval ratio and pinnule size along the primary pinna have been detected, and the relationship between these variables, expressed by the braching ratio, has a determined pattern. None of the reconstructions up to date noted these variations that are extremely important to define the shape of the primary pinna and differentiate between mature and juvenile primary pinnae, and also provide insight to their growth pattern.

The pinnules can’t be properly seen in the reconstructions; only Poyato-Ariza & Buscalioni (2016) illustrate a detail of a secondary pinna with pinnules. The distribution of pinnules along the secondary pinna is opposite and becomes alternate distally, similarly to our observations. Insertion angles in the reconstruction proposed in Poyato-Ariza & Buscalioni (2016) show a similar pattern to that observed in this work, although the measurements differ. The change in pinnule morphology is also represented in and is commented by Alvin (1971). Pinnule size in Poyato-Ariza & Buscalioni (2016) is reduced distally at a stable rate, there are no “steps” like the ones herby identified.

Although the reconstruction proposed in Poyato-Ariza & Buscalioni (2016) is based on the same material used in this work, the differences are important. A better understanding of the architecture, based on morphometrical data, allows for a more precise description of the general morphology of the plant, and to understand how this morphology is obtained.
